# *Erwinia amylovora* psychrotrophic adaptations: evidence of pathogenic potential and survival at temperate and low environmental temperatures

**DOI:** 10.7717/peerj.3931

**Published:** 2017-10-26

**Authors:** Ricardo D. Santander, Elena G. Biosca

**Affiliations:** Department of Microbiology and Ecology, Universitat de València, Burjassot, Spain

**Keywords:** Fire blight, EPS, Biofilms, Oxidative stress, Siderophores, VBNC, Motility, Starvation, Virulence

## Abstract

The fire blight pathogen *Erwinia amylovora* can be considered a psychrotrophic bacterial species since it can grow at temperatures ranging from 4 °C to 37 °C, with an optimum of 28 °C. In many plant pathogens the expression of virulence determinants is restricted to a certain range of temperatures. In the case of *E. amylovora,* temperatures above 18 °C are required for blossom blight epidemics under field conditions. Moreover, this bacterium is able to infect a variety of host tissues/organs apart from flowers, but it is still unknown how environmental temperatures, especially those below 18 °C, affect the pathogen ability to cause fire blight disease symptoms in such tissues/organs. There is also scarce information on how temperatures below 18 °C affect the *E. amylovora* starvation-survival responses, which might determine its persistence in the environment and probably contribute to the seasonal development of fire blight disease, as occurs in other pathogens. To characterize the virulence and survival of *E. amylovora* at temperate and low temperatures, we evaluated the effect of three temperatures (4 °C, 14 °C, 28 °C) on symptom development, and on different parameters linked to starvation and virulence. *E. amylovora* was pathogenic at the three assayed temperatures, with a slow-down of symptom development correlating with colder temperatures and slower growth rates. Siderophore secretion and motility also decreased in parallel to incubation temperatures. However, production of the exopolysaccharides amylovoran and levan was enhanced at 4 °C and 14 °C, respectively. Similarly, biofilm formation, and oxidative stress resistance were improved at 14 °C, with this temperature also favoring the maintenance of culturability, together with a reduction in cell size and the acquisition of rounded shapes in *E. amylovora* cells subjected to long-term starvation. However, starvation at 28 °C and 4 °C induced an enhanced viable but nonculturable (VBNC) response (to a lesser extent at 4 °C). This work reveals *E. amylovora* as a highly adaptable pathogen that retains its pathogenic potential even at the minimal growth temperatures, with an improved exopolysaccharide synthesis, biofilm formation or oxidative stress resistance at 14 °C, with respect to the optimal growth temperature (28 °C). Finally, our results also demonstrate the thermal modulation of starvation responses in *E. amylovora,* suggesting that the starvation-survival and the VBNC states are part of its life cycle. These results confirm the particular psychrotrophic adaptations of *E. amylovora*, revealing its pathogenic potential and survival at temperate and low environmental temperatures, which have probably contributed to its successful spread to countries with different climates. This knowledge might improve integrated control measures against fire blight.

## Introduction

*Erwinia amylovora* is a Gram-negative bacterium that causes fire blight, a destructive plant disease affecting economically important fruit trees such as pear and apple, as well as ornamental plants of the family *Rosaceae*. Among the environmental factors affecting the development of fire blight, temperature is a critical one, directly affecting bacterial growth, the phenologic development of the host (and interactions among these factors), and determining the presence of vectors responsible for the spread of the pathogen (Thomson, 2000; Van der Zwet, Orolaza-Halbrendt & Zeller, 2012).

Temperature ranges allowing bacterial growth are usually linked to the ecology of each bacterial species, with the ability of bacteria to adapt and/or tolerate certain temperatures directly conditioning their pathogenicity, virulence and/or niches (Jay, 1998; Scherf, Milling & Allen, 2010; Burbank & Stenger, 2016; Du Raan, Coutinho & Van der Waals, 2016; Morris et al., 2016). Compared to other phytopathogens (Du Raan, Coutinho & Van der Waals, 2016; Burbank & Stenger, 2016), many of them mesophilic, *E. amylovora* possesses an unusually wide range of growth temperatures (4 °C–37 °C), the optimal temperature being 28 °C (Van der Zwet, Orolaza-Halbrendt & Zeller, 2012). It is for this reason that this pathogen can be considered a psychrotrophic microorganism (i.e., able to grow at low temperatures but with optimal and maximal growth temperatures above 15 °C and 20 °C, respectively) (Moyer & Morita, 2007). However, how this characteristic is connected to the *E. amylovora* life cycle is still non-fully understood.

The ability to cause disease of many pathogens is restricted to a certain range of temperatures, and suppressed or very reduced at different ones (Konkel & Tilly, 2000; Smirnova et al., 2001; Case et al., 2011; Kimes et al., 2012; Guijarro et al., 2015; Du Raan, Coutinho & Van der Waals, 2016). In plant pathogenic bacteria, the temperatures enhancing the deployment of virulence factors use to be lower than the optimal for growth (16–24 °C), coinciding with the ones favoring the formation of water aerosols or films near/on plant surfaces (which are usually required for efficient infection), or with the ones of water films (Smirnova et al., 2001). In the case of *E. amylovora*, a temperature of 18 °C seems required for blossom blight epidemics to occur under field conditions (Thomson, 2000; Van der Zwet, Orolaza-Halbrendt & Zeller, 2012), and rain and/or heavy dew at the end of warm periods promote infection events in the field (Johnson & Stockwell, 1998). Moreover, at 18 °C, the expression of pathogenicity and other function genes is enhanced with respect to higher temperatures (Wei, Sneath & Beer, 1992; Goyer & Ullrich, 2006). However, despite flowers are the main organ for the *E. amylovora* entry into the host, other infection routes (e.g., via stomata, wounds caused by hail, wind, insects, etc.) contribute to the phenomenology of fire blight epidemics, which difficult disease control (Thomson, 2000; Billing, 2011; Van der Zwet, Orolaza-Halbrendt & Zeller, 2012). In this regard, there is still scarce information on the *E. amylovora* ability to infect susceptible tissues/organs other than flowers at temperatures below 18 °C, or about how such temperatures may affect the secretion of virulence factors.

The effect of temperature on *E. amylovora* (and other pathogens) varies depending on the nutritional state of cells. When inside the host, or as an epiphyte on floral organs, nutrient availability together with appropriate environmental temperatures favor bacterial cell multiplication. However, when *E. amylovora* experiences nutrient limitation (e.g., on plant surfaces, rainwater, soil, vector insects, etc.) this stress induces, in a temperature dependent manner, growth arrest, morphological changes and the development of a starvation-survival response with subpopulations of cells entering the viable but nonculturable (VBNC) state (Santander et al., 2012; Santander, Oliver & Biosca, 2014; Ordax et al., 2015).

The VBNC state was firstly described in *E. amylovora* in response to copper stress (Ordax et al., 2006). However, regardless of the VBNC-inducing stress (e.g., copper, starvation, chlorine, etc.), VBNC cells are able to recover culturability under certain conditions (Ordax et al., 2009; Santander et al., 2012; Ordax et al., 2015), thus constituting potential and hard-to-detect inoculum sources of the pathogen in the environment.

The starvation-survival and the VBNC state are bacterial strategies to face harsh nutrient and temperature conditions, and may have an impact on the ecology of many pathogens and/or the phenomenology of the disease they cause (Biosca et al., 1996; Armada et al., 2003; Caruso et al., 2005; Vattakaven et al., 2006; Lutz et al., 2013; Ayrapetyan et al., 2015). However, the role of such strategies in the *E. amylovora* life cycle has not been well established, and little is known about the effects of environmental temperatures below 18 °C on the pathogen responses to starvation, or how such responses might be linked to its life cycle.

We hypothesize that the *E. amylovora* psychrotrophic adaptations may have repercussions on its ability to infect susceptible plant material and starvation responses at environmental temperatures below 18 °C. In this work, we investigated the effect of warm (28 °C), temperate (14 °C) and low (4 °C) temperatures on *E. amylovora* virulence and survival under natural starvation conditions, as well as on different factors related to virulence and/or environmental survival. Our results provide new knowledge on scarcely studied aspects of the *E. amylovora* life cycle, such as the temperature-dependent modulation of virulence and starvation responses which have probably contributed to the successful spread of this bacterial species to numerous countries worldwide with different climate areas (EPPO, 2017).

## Material and Methods

### Biosafety approval

The Universitat de València granted Biosafety approval to carry out the study within its facilities.

### Bacterial strains and culture conditions

Five *E. amylovora* strains from different hosts and geographical origins frequently used in studies on fire blight were employed in this work: Ea 1/79 (Falkenstein et al., 1988), Ea 1189 (Burse, Weingart & Ullrich, 2004), CFBP 1430 (Paulin & Samson, 1973), NCPPB 2080 (Giorgi & Scortichini, 2005) and ATTC 49946 (Sebaihia et al., 2010). Unless otherwise specified, bacterial cultures were grown on LB agar (LBA) plates or in liquid LB at 28 °C with shaking (150 rpm). Strains were cryopreserved at −80 °C in 25% glycerol.

### Virulence

The virulence of the five *E. amylovora* strains included in this study was tested using green loquats (cv Tanaka). One-off assays were additionally performed on green pears (cv Devoe) with the reference strain CFBP 1430, as well as on pear shoots (cv. Conference) with strains CFBP 1430 and ATCC 49946. To reduce fungal growth during prolonged incubation periods, particularly at low temperatures, fruits were first surface disinfected with 2% sodium hypochlorite as previously described (Santander et al., 2014) and then treated with 21.6 mg L^−1^ natamycin (Nataproq-G; Proquiga Biotech, Bergondo, Spain) (Pedersen, 1992) for 5 min. This compound is a broad-spectrum fungicide, with low toxicity, used in multiple applications (Pedersen, 1992; Pengfei, Junxing & Shaobo, 2013; Biosca et al., 2016). Based in our previous experience, applying natamycin to immature fruits prior to their inoculation efficiently delays fungal growth, allowing prolonged incubations without fungal contaminations. Natamycin treated fruits were let to dry in aseptic conditions under the hood, and then wound-inoculated with 10^3^ CFU of *E. amylovora* per wound according to Santander et al. (2014), incubated in a wet chamber at 4 °C, 14 °C and 28 °C, and periodically monitored for fire blight symptoms development. One-off pear shoot assays were performed according to Ordax et al. (2009), at 28 °C and 14 °C, with four shoots per strain and temperature, incubated in a climatic chamber. *E. amylovora* was re-isolated from symptomatic fruits and shoots and identified by specific PCR as previously described (Santander et al., 2012). Ten fruits were inoculated per strain and temperature, and the experiment was performed in two independent repeats.

### Growth curves

Growth curves were performed at 4 °C, 14 °C and 28 °C in LB broth under static conditions. For this purpose, overnight cultures in LB were adjusted to an OD_600_ nm of 1.0 (ca. 2 10^9^ CFU mL^−1^), diluted 1:50 into fresh medium and incubated in parallel under static conditions at the above-mentioned temperatures. Aliquots of 0.5 mL were periodically taken from cultures and the OD_600_ nm measured. This experiment was performed in duplicate, per strain and temperature.

### Siderophore detection

To induce the biosynthesis/secretion of siderophores, AB minimal medium (Chilton et al., 1974) containing 0.05 g L^−1^ nicotinic acid (ABN) to allow *E. amylovora* growth (Starr & Mandel, 1950) and lacking FeSO_4_ (ABN-Fe) was employed. *E. amylovora* overnight cultures in LB were pelleted, washed twice, resuspended in fresh ABN-Fe medium to an OD_600_ nm of 0.3, and incubated in parallel at 28°C, 14 °C and 4 °C. Siderophore levels were quantified after 0, 1, 3 and 7 days post-inoculation (dpi) by mixing 0.4 mL of supernatant with 0.3 mL of a fresh solution of 0.12 M FeCl_3_ in 5 mM HCl (Holzberg & Artis, 1983). The abundance of siderophores in culture supernatants was spectrophotometrically determined at A_425_ nm, and normalized to the OD_600_ nm of the analyzed cultures. This experiment was performed in duplicate, in two independent experiments.

### Motility assays in soft agar

Swimming motility was evaluated using TG (1% tryptone, 0.5% glucose, pH 7.0) soft agar plates (0.3% agar). Briefly, a sterile toothpick previously dipped into an overnight culture of the *E. amylovora* strain to test was used to inoculate soft agar plates, which were sealed with parafilm and incubated at 4 °C, 14 °C and 28 °C. This assay was performed in two independent experiments, each with three technical repeats.

### EPS relative quantification

To induce the production of the main *E. amylovora* EPSs amylovoran and levan, overnight cultures were washed and diluted to an OD_600_ nm of 0.4 in LB plus 1% sorbitol (LB Sor) or 5% sucrose (LB Suc), respectively (Geider, 2000). Cultures were incubated at 4 °C, 14 °C and 28 °C and EPS contents in supernatants were measured at times 1, 3 and 7 dpi.

Amylovoran was measured (OD_600_ nm) after a 10 min reaction of 0.8 mL LB Sor supernatants with 40 µL of 50 mg mL^−1^ cetylpyridinium chloride (CPC) (Ordax et al., 2010). Levan production was measured as an increase in turbidity (OD_580_ nm) in LB Suc culture supernatants (Ordax et al., 2010).

Amylovoran and levan levels were normalized to the OD_600_ nm of the analyzed culture in each case (Santander et al., 2014). This assay was performed in duplicate in two independent experiments.

### Biofilm quantification by a microtiter assay

*E. amylovora* biofilms were quantitated using a microtiter assay based on Chelvam, Chai & Thong (2014). To this aim, *E. amylovora* overnight cultures were diluted to an OD_600_ nm of 0.5 into 0.5× LB broth, and transferred (160 µL per well) to polystyrene (surface-treated, hydrophylic) Nunc™ MicroWell™ 96-well microplates (Thermo Scientific, Waltham, MA, USA). Sterility controls consisting of non-inoculated 0.5× LB were included in each assay. Inoculated plates were incubated in parallel at 4 °C, 14 °C and 28 °C for 48 h. After this period, the plates were left to reach room temperature for 10 min, and planktonic cells and medium were removed by inversion, gentle tapping and incubation upside down for 5 min, inside a biosafety cabinet. Then, biofilms were heat-fixed in a Pasteur oven at 80 °C for 30 min and let to cool down to room temperature. Afterwards, 220 µL of 1% crystal violet (CV) were added to each well and incubated for 15 min at room temperature. The CV was removed by inversion, and the plates were thoroughly rinsed with distilled water and let to dry upside down inside the hood. Biofilm formation was determined spectrophotometrically (A_600_ nm) with a plate reader (FLUOstar OPTIMA; BMG Labtech, Ortenberg, Germany), after solubilizing the CV from biofilms with an 8:2 mixture of absolute ethanol and acetone (220 µL per well) for 20 min. This assay was carried out with, at least, seven replicates per strain and temperature, in two independent experiments.

### Oxidative stress resistance

Oxidative stress sensitivity at 4 °C, 14 °C and 28 °C was assessed by an agar dilution assay, based on Wiegand, Hilpert & Hancock (2008). Briefly, plates of LBA (control) and LBA + 0.75 mM H_2_O_2_ were prepared and let to solidify under the hood for 30 min. The H_2_O_2_ concentration was selected based on preliminary assays. *E. amylovora* overnight cultures in LB were adjusted to an OD_600_ nm of 1.0 (ca. 10^9^ CFU mL^−1^), serially tenfold diluted in sterile saline, and 5 µL drops of each dilution (from 10^−1^ to 10^−6^) were plated on the above-mentioned media. Plates were then sealed and incubated in parallel at 28 °C, 14 °C and 4 °C for 6, 18 and 41 days, respectively. The minimal dilution at which each strain grew on each medium at each temperature was recorded. This assay was performed in two independent repeats.

### Microcosms preparation, monitoring of population dynamics and morphology analysis of starved cells

Oligotrophic water microcosms were prepared as previously described (Santander et al., 2012), inoculated with *E. amylovora* at a final density of ca. 2 10^7^ CFU mL^−1^, and incubated in parallel at 4 °C, 14 °C and 28 °C. Culturable cell populations at different periods were determined by drop-plate on LBA, as described elsewhere (Santander et al., 2012). Viable and total cell population dynamics were monitored by flow cytometry, using a BD FACSVerse™ flow cytometer (BD Biosciences, San Jose, CA, USA), with 488 nm excitation. To this aim, microcosm aliquots were taken at different intervals throughout a 122-day period, and stained with the components of the Baclight Live/Dead viability kit (Life Technologies), according to the manufacturer’s instructions. Red and green fluorescence were measured with 700/54 and 527/32 filters, respectively, the trigger was set on FSC (forward scatter), and data were analyzed using the BD FACSuite™ software (BD Biosciences, San Jose, CA, USA). Each strain was inoculated in three independent microcosms per temperature.

Morphological changes occurring in *E. amylovora* cells starved in water microcosms were monitored by epifluorescence microscopy similar to Álvarez, López & Biosca (2008). Briefly, 20 µL microcosm aliquots were taken at times 0 and 122 dpi, fixed on a glass slide and stained for 10 min with 12 mM SYTO9 (Life Technologies, Carlsbad, CA, USA). Afterwards, the stained cells were covered with a cover slip and photographed at a magnification of ×1,000, using a digital camera adapted to a Nikon Eclipse E800 epifluorescence microscope, and the automatic camera tamer software ACT-1. Microscope images were thresholded to create binary images with IMAGEJ software (Schneider, Rasband & Eliceiri, 2012), and the parameters Feret diameter, area and circularity were measured to determine possible changes in cell size and shape due to starvation and/or temperature.

### Statistical analysis

Prior to statistical analysis a log transformation of culturable, viable, and total cell counts was performed. In general, differences between means were determined by analysis of variance (ANOVA). The factors compared were temperature and strain, although the time factor was also included in certain analyses. In some experiments replicate means were compared using Bonferroni post-tests. *P*-values ≤0.05 were considered significant.

## Results & Discussion

### *E. amylovora* remains pathogenic even at 4 °C, with virulence, growth rates, siderophore secretion and motility decreasing in parallel to incubation temperature

**Figure 1:**
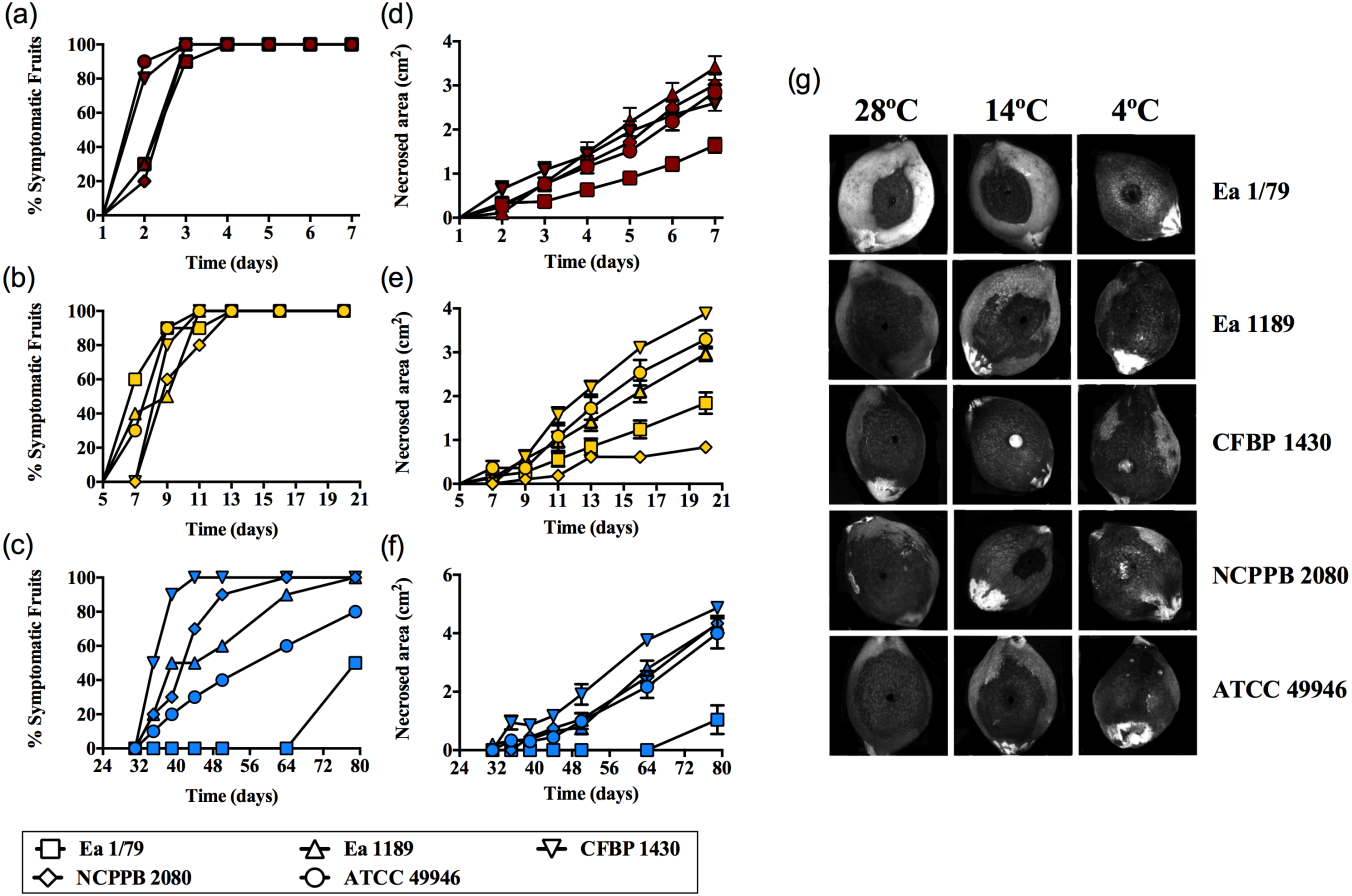
*E. amylovora* virulence assays in immature loquats (cv. Tanaka) at 28 °C (A, D), 14 °C (B, E) and 4 °C (C, F). Percentage of symptomatic fruits (total values) over time (A–C) and expansion of necrotic lesions over time (D–F). Representative pictures of fruits showing fire blight symptoms at the end of the experimental period (28 °C, 7 dpi; 14 °C, 20 dpi; 4 °C, 79 dpi) (G). These data show the results from a representative experiment performed with ten replicates and repeated in two independent experiments with equivalent results.

Results corresponding to virulence assays at 4 °C, 14 °C and 28 °C in green loquats are shown in Figs. 1A–1G. All the *E. amylovora* assayed strains were able to cause typical fruit blight symptoms (necrosis and exudates) at the three tested temperatures. However, the onset of symptoms and the progression of necrosis throughout time were strongly dependent on temperature (*p* < 0.001). Fruit blight symptoms developed earlier at 28 °C (2 dpi) (Fig. 1A), followed by 14 °C (7–9 dpi) (Fig. 1B) and 4 °C (35–79 dpi) (Fig. 1C). In the same way, the fastest necrosis expansion over time was observed at 28 °C, followed by 14 °C and 4 °C (Figs. 1D–1F). These results were similarly reproduced in virulence assays with green pears (cv. Devoe) inoculated with the *E. amylovora* strain CFBP 1430 (Fig. S1). In the case of one-off assays with pear shoots (*P. communis* var. Conference), symptoms also developed in 7–9 dpi at 14 °C, and at 2–3 dpi at 28°C.

It is noteworthy that *E. amylovora* retained its pathogenicity at 14 °C and even at 4 °C. In this regard, despite flowers are the main *E. amylovora* entry site into the host, and a temperature of 18 °C is required for blossom blight epidemics development (Thomson, 2000; Van der Zwet, Orolaza-Halbrendt & Zeller, 2012), our results shed light on the potential ability of this pathogen to initiate infections and cause fire blight symptoms in other tissues, at lower environmental temperatures. This is in agreement with the reported progression of fire blight symptoms in perennial pear branches during winter in Israel, following autumn infections (Shtienberg et al., 2015). Moreover, the slow progression of symptom development at such temperatures might allow latent infections to keep hidden until higher environmental temperatures favor symptom emergence, although field studies are required to confirm this hypothesis.

The retention of pathogenicity at a wide range of environmental temperature is not a common behavior among pathogenic bacteria. As an example, the ability to cause disease and/or the production of some virulence factors in a variety of bacterial phytopathogens (e.g., species of *Dickeya, Pectobacterium, Pseudomonas, Ralstonia,* and *Rhizobium*) is restricted to a certain temperature range (Serfontein et al., 1991; Durand et al., 2000; Goyer & Ullrich, 2006; Bocsanczy et al., 2014; Du Raan, Coutinho & Van der Waals, 2016). This is due to the repression of virulence and/or pathogenicity genes by temperatures above or below this range (Konkel & Tilly, 2000; Smirnova et al., 2001).

Our results show *E. amylovora* as a highly adaptable pathogen to a range of environmental temperatures, more active at 28 °C, but retaining its pathogenic potential at lower temperatures (even at 14 °C and 4 °C). In this sense, the wide temperature range at which *E. amylovora* is potentially pathogenic is probably a crucial factor allowing the pathogen to successfully colonize new areas with diverse climate conditions.

**Figure 2:**
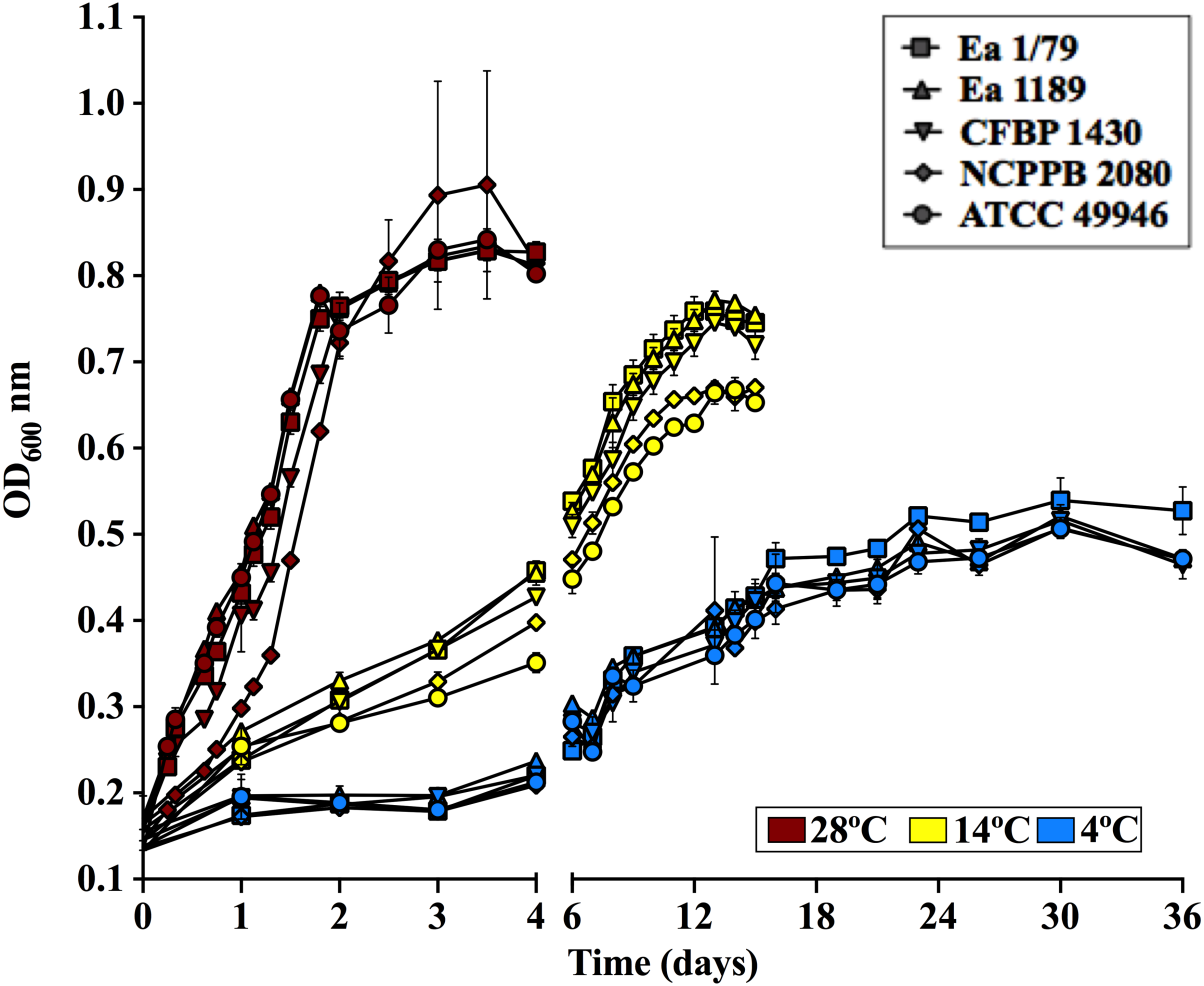
*E. amylovora* growth curves in LB at 28 °C, 14 °C and 4 °C under static conditions. Vertical lines indicate the SD. These data show the results from a representative experiment performed in duplicate.

**Figure 3:**
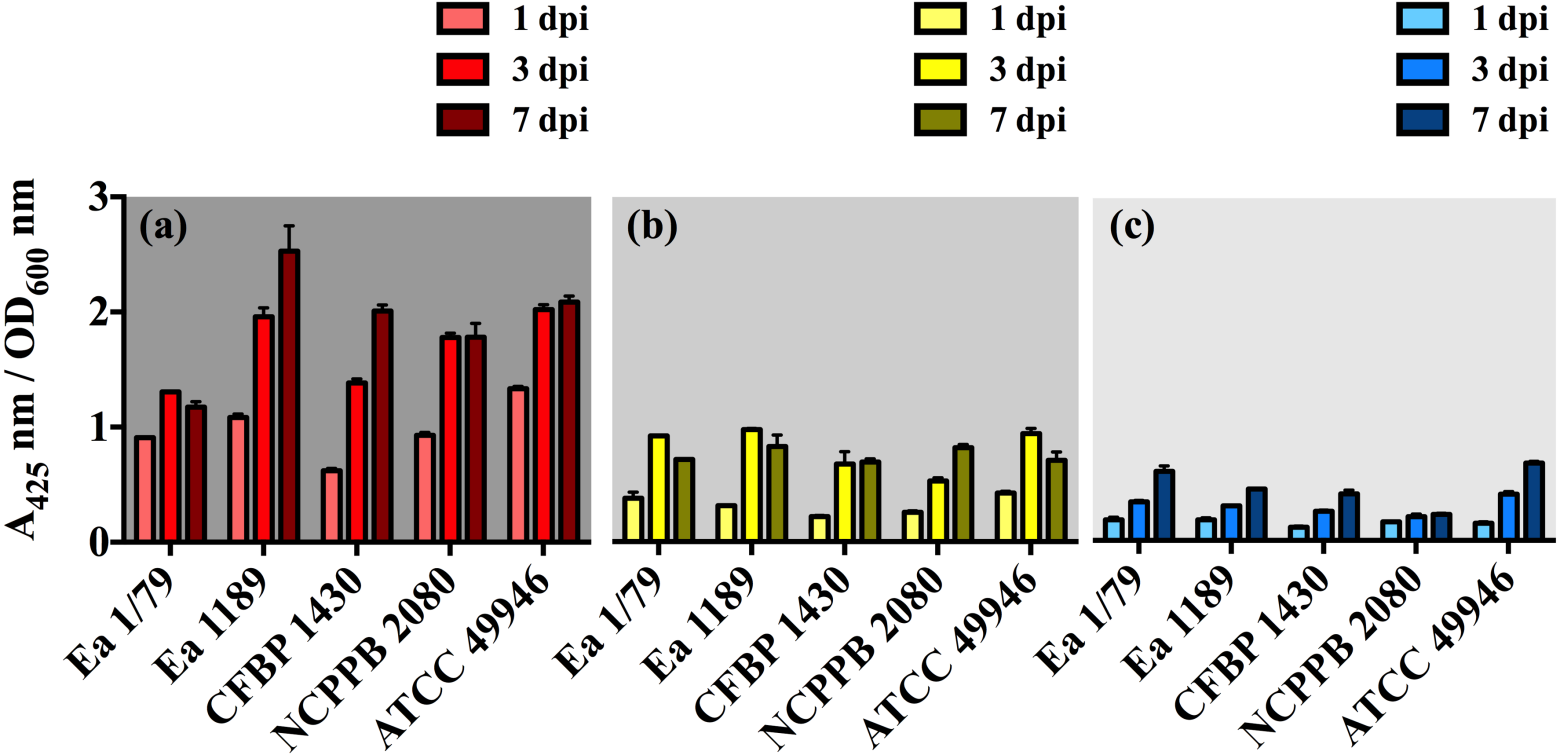
*E. amylovora* siderophore production at 28 °C (A), 14 °C (B) and 4 °C (C) in minimal medium ABN-Fe after 1, 3 and 7 dpi. Vertical lines mark the SD. The two-way ANOVA analysis of these results showed that differences of siderophore production among the three temperatures were very significant (*p* < 0.0001). These data show the results from a representative experiment performed in duplicate and repeated twice, with similar results.

**Figure 4:**
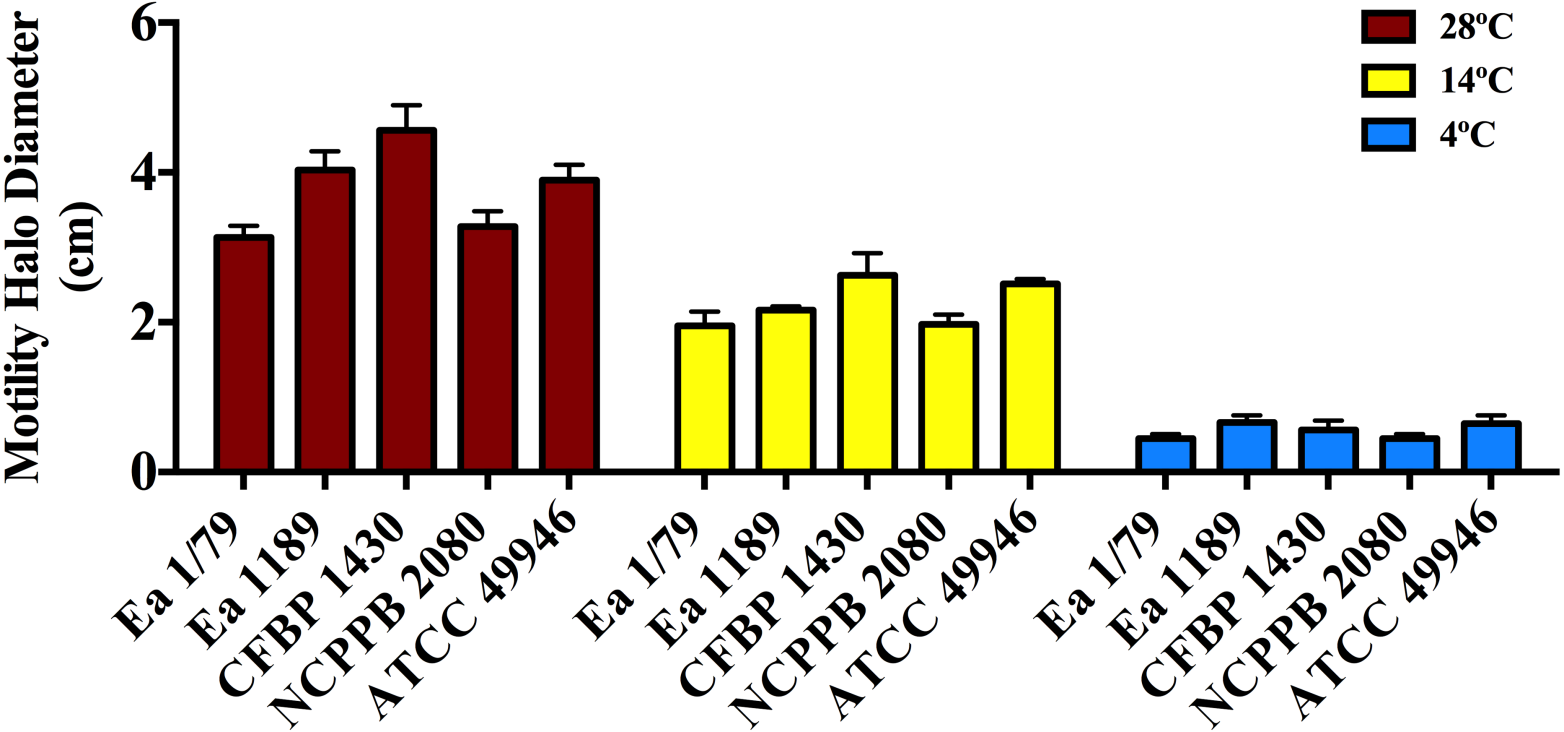
Motility of *E. amylovora* in soft agar plates at 28 °C, 14 °C and 4 °C after 2 days of incubation. Vertical lines mark the SD. Swimming motility differences at the three temperatures assayed were very significant (*p* < 0.001), regardless of the tested strain. These data are from a representative experiment of two, performed in triplicate and with similar results.

The effect of incubation temperature on symptom development was similar to that observed in growth rates (Fig. 2), siderophores secretion (Figs. 3A–3C) and motility (Fig. 4). Coinciding with fire blight symptom development, these virulence determinants reached lower values the lower the assayed incubation temperature was.

Regarding growth rates, some virulence factors in *E. amylovora* are regulated in a cell population dependent manner via *quorum sensing* (Molina et al., 2005). Therefore, it is reasonable that fruit blight symptoms appear and develop faster at temperatures allowing *E. amylovora* to reach appropriate cell densities in shorter periods, which correlates with the higher growth rates observed at 28°C, followed by 14 °C and 4 °C (Fig. 2). Interestingly, Pusey & Curry (2004) demonstrated that the *E. amylovora* proliferation on stigma surfaces of crab apple flowers only occurs at temperatures above 12 °C, with temperatures ranging from 20 °C to 32 °C allowing the pathogen to reach cell densities greater than those of antagonistic bacteria. This fact, among others, might explain why blossom blight in field is often observed at temperatures above 18 °C (Van der Zwet, Orolaza-Halbrendt & Zeller, 2012). However, growth curves and virulence assays in immature fruits as well as in pear shoots indicate that *E. amylovora* not only grows at temperatures below 18 °C, but is also able to cause fire blight symptoms once it reaches susceptible host tissues.

With respect to siderophores, these iron chelating compounds have long been recognized as important virulence factors in *E. amylovora*, contributing to iron acquisition inside the host and survival under oxidative stress (Dellagi et al., 1998; Dellagi et al., 1999; Venisse, Gullner & Brisset, 2001; Venisse et al., 2003). In this study, we identified a decrease in siderophore production occurring in parallel with the diminution of incubation temperature, with the relative levels of these iron chelating compounds at 28 °C being 2.5 (*p* < 0.001) and 5.4 (*p* < 0.001) times greater than those determined at 14 °C and 4 °C, respectively (Figs. 3A–3C). This might be related to the diminished iron requirements as temperature decreases, as a consequence of a slowed down metabolism. The lower siderophore production, together with the reduced virulence as temperature decreased, might also support the important role of these iron-uptake molecules as *E. amylovora* virulence factors.

Motility in *E. amylovora* is necessary for full virulence, aiding bacterial cells to reach natural openings in apple blossoms (Bayot & Ries, 1986) or other plant organs (Cesbron et al., 2006). This phenotypic trait is modulated by temperature, pH and other environmental factors (Raymundo & Ries, 1980; Goyer & Ullrich, 2006). Once inside host tissues, *E. amylovora* cells lose flagella (Raymundo & Ries, 1981; Cesbron et al., 2006), avoiding the elicitation of plant defense responses by flagellins (Cesbron et al., 2006; Holtappels et al., 2015). However, non-motile cells from infected tissues can recover their motility if water films on plant surfaces are formed (Raymundo & Ries, 1981). Furthermore, cells expressing flagella can remain motile even under starvation conditions (Santander, Oliver & Biosca, 2014) and for variable periods, also depending on incubation temperatures (Raymundo & Ries, 1981). This probably makes it easier to reach new host entry sites and/or other nutrient sources.

As shown in Fig. 4, all the assayed *E. amylovora* strains were able to move throughout soft agar regardless of the tested temperature, with the greatest motility observed at 28 °C, followed by 14 °C and 4 °C. According to our results, *E. amylovora* has the potential to move towards new host entry sites even at suboptimal environmental growth temperatures, however chemotactic responses decrease above 28 °C and below 20 °C (Raymundo & Ries, 1980). This could suggest reduced chances for bacterial cells to locate nectaries at temperatures below 20 °C. Nevertheless, motility is repressed after entering into host tissues (Raymundo & Ries, 1981; Cesbron et al., 2006) and would not be necessary, for example when *E. amylovora* cells enter into the host through wounds, with these bacterial cells having the potential to invade host tissues even at minimal growth temperatures.

### EPS production, biofilm formation and oxidative stress resistance are enhanced at temperatures below the optimal for growth

Two important virulence/pathogenicity factors in the fire blight pathogen and other phytopathogenic bacteria are the production of EPSs and the formation of biofilms (Koczan et al., 2009; Koczan et al., 2011; Van der Zwet, Orolaza-Halbrendt & Zeller, 2012; Piqué et al., 2015; Castiblanco & Sundin, 2016). EPSs contribute to pathogen spread and host colonization, by obstruction of the plant vascular system and masking the bacterial cell surface elicitors of plant defenses (Geider, 2000; Maes et al., 2001; Venisse, Gullner & Brisset, 2001; Ordax et al., 2015). Moreover, they might favor pathogen survival in the environment, acting as alternative carbon sources during starvation periods, and as a barrier against desiccation, bacteriophages and toxic compounds such as copper (Geider, 2000; Jock, Langlotz & Geider, 2005; Ordax et al., 2010; Van der Zwet, Orolaza-Halbrendt & Zeller, 2012; Born et al., 2014). With regard to biofilms, their role during the systemic invasion of plant hosts has been reported (Koczan et al., 2009; Koczan et al., 2011), as well as their relevance for the survival of the pathogen on plant surfaces and vectors (Ordax et al., 2009; Ordax et al., 2015).

**Figure 5:**
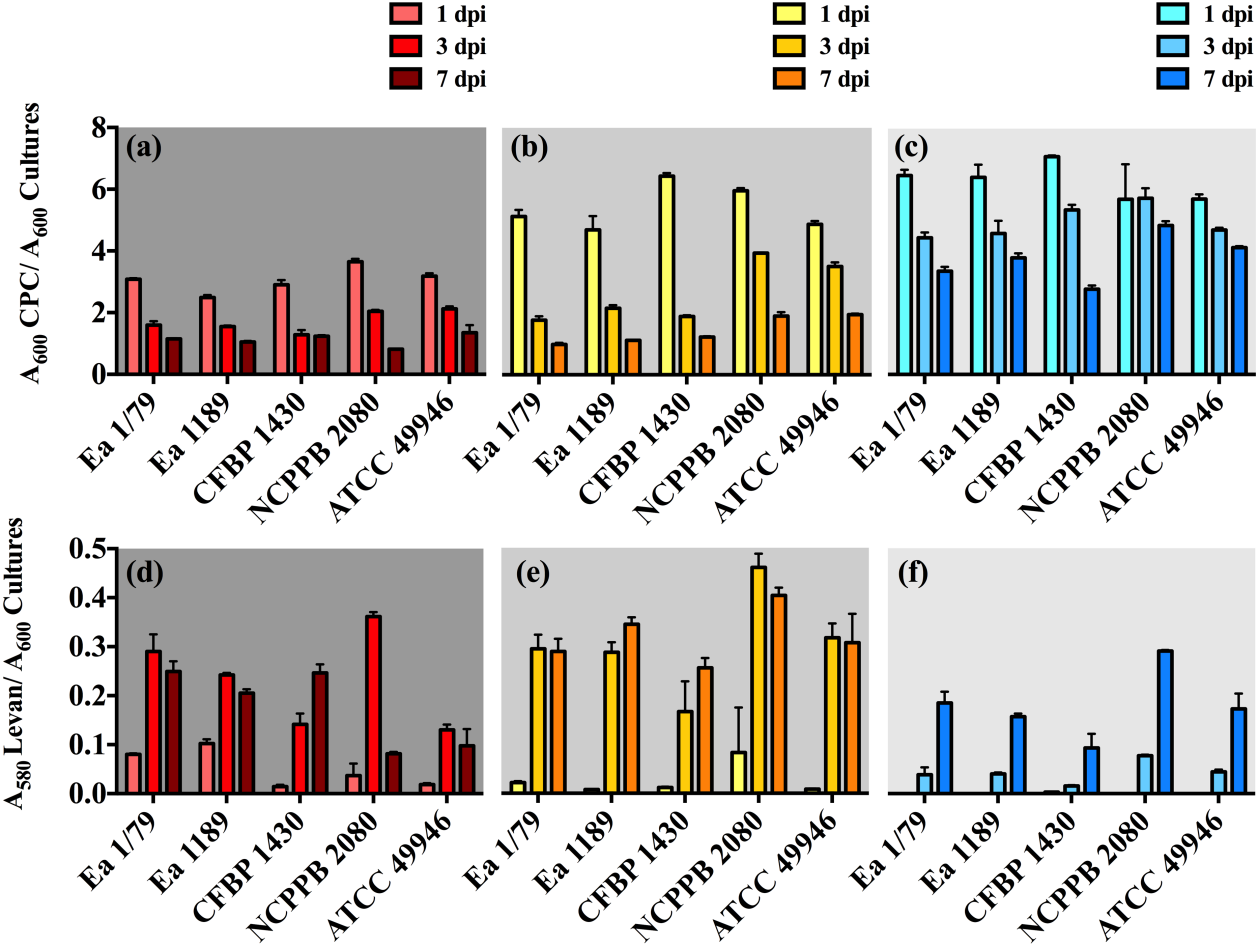
Relative production of amylovoran (A–C) and levan (D–F) by *E. amylovora* at 28 °C (A, D), 14 °C (B, E) and 4 °C (C, F). Vertical lines denote the SD. A two-way ANOVA analysis of data revealed that differences in EPS production at 28 °C, 14 °C and 4 °C were very significant (*p* < 0.0001), regardless of the assayed strain. These data are from a representative experiment performed in duplicate, being similar to those obtained in a second independent experiment.

**Figure 6:**
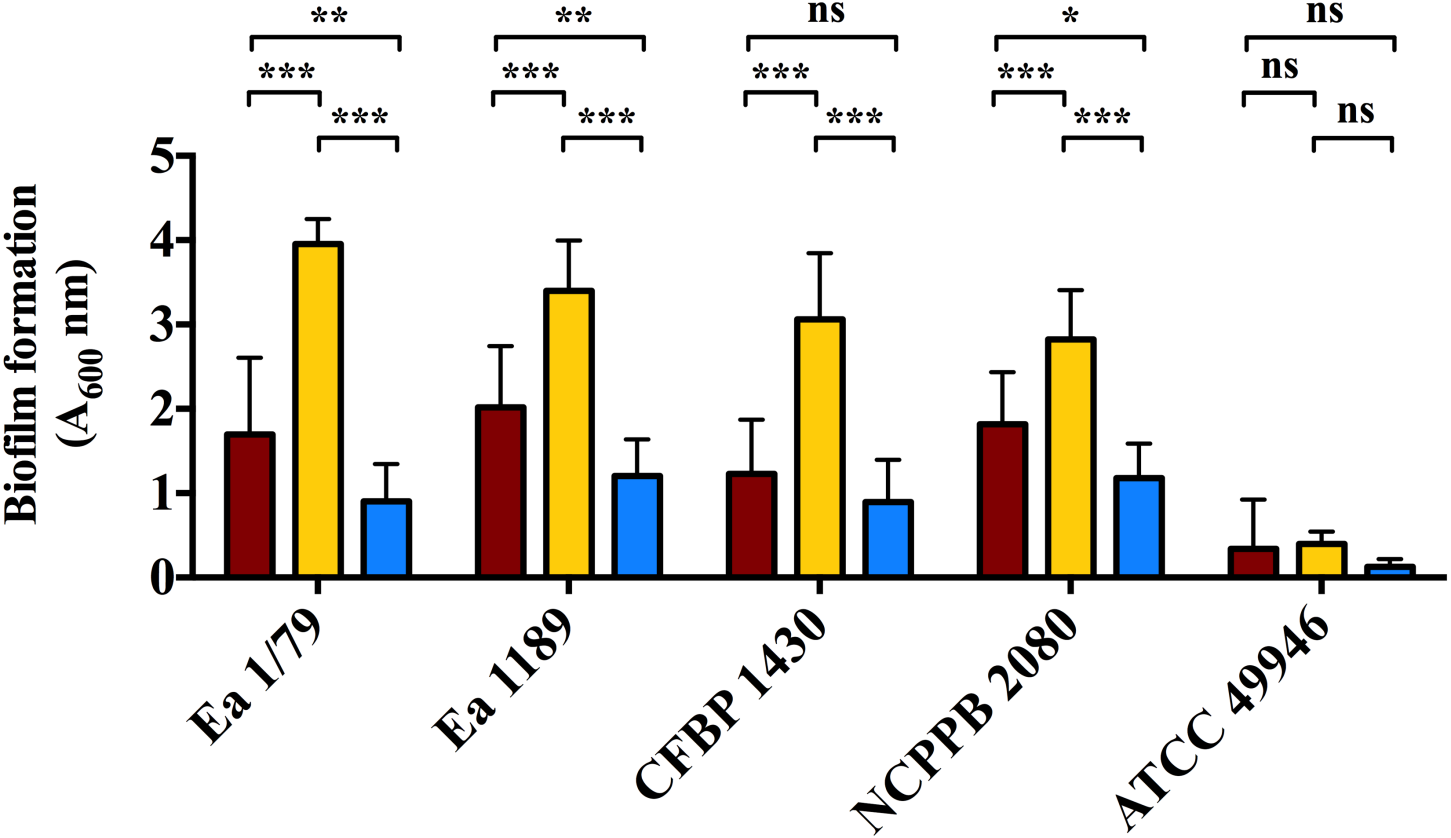
Quantification of biofilm formation by *E. amylovora* after 48 h of incubation at 28 °C (red), 14 °C (yellow) and 4 °C (blue). Vertical lines indicate the SD. Asterisks above horizontal lines denote statistically significant differences between the two signaled columns. *, *p* < 0.05; **, *p* < 0.01; ***, *p* < 0.001; ns, non-significant differences. These data are from a representative experiment carried out, at least, with seven replicates per strain and temperature. This experiment was repeated with the same number of replicates, showing similar results.

In our work, we provide for the first time data on the ability of *E. amylovora* to produce EPSs (Figs. 5A–5F) and biofilms (Fig. 6) at temperatures ranging from 4 °C to 28 °C, with these virulence factors being more intensely induced at temperatures below the optimal for growth (*p* < 0.0001).

Our results also revealed an interesting differentiated temperature-dependent modulation of the two main EPSs in *E. amylovora,* amylovoran and levan (Figs. 5A–5F). Amylovoran was more intensively produced at 4 °C, followed by 14 °C and 28 °C, reaching maximum levels at 1 dpi (Figs. 5A–5C). Levan production, however, increased over time regardless of the assayed temperature, with levels of this EPS reaching maximum values at 3 and 7 dpi, and being greater at 14 °C, than at 28 °C or 4 °C (Figs. 5D–5F). These results might suggest a not yet described role for EPSs in *E. amylovora* facilitating growth at low temperatures and/or enhancing cold tolerance, as described in bacteria from cold environments (Nichols et al., 2005; Chrismas et al., 2016).

In some cases, a reduction in EPS levels (mainly amylovoran) over time was observed (Figs. 5A–5F). This phenomenon has been linked to their use as alternative carbon sources under nutrient limiting conditions (Ordax et al., 2010). In our case, transitory nutrient starvation of *E. amylovora* cells due to their incubation for a week under static conditions could explain the results obtained.

Analogous to levan production, biofilm formation in all the assayed *E. amylovora* strains was enhanced at 14 °C, followed by 28 °C and 4°C (Fig. 6). This demonstrates the potential of *E. amylovora* to form biofilms in plant tissues at 14 °C and even at 4 °C, suggesting that the systemic invasion of host plants is possible even at very low temperatures. Furthermore, the need of both amylovoran and levan for proper biofilm development in *E. amylovora* has been described (Koczan et al., 2009). Accordingly, the observed differences in the thermal modulation patterns of these two EPSs might indicate that their degree of contribution to biofilm formation might also vary depending on environmental temperatures.

Taken together the above-mentioned virulence related results, from assays with five different strains of the pathogen from different geographical areas, demonstrate the ability of *E. amylovora* to cause fire blight symptoms at 28 °C, 14 °C, and 4 °C. However, the apparent discrepancy between virulence results and the differential production of virulence determinants at each temperature, suggest the complex nature of *E. amylovora* interactions with its hosts, as well as the potential influence of other ecological abiotic and biotic conditions, such as temperature, surrounding environmental microbiota, etc.

**Figure 7:**
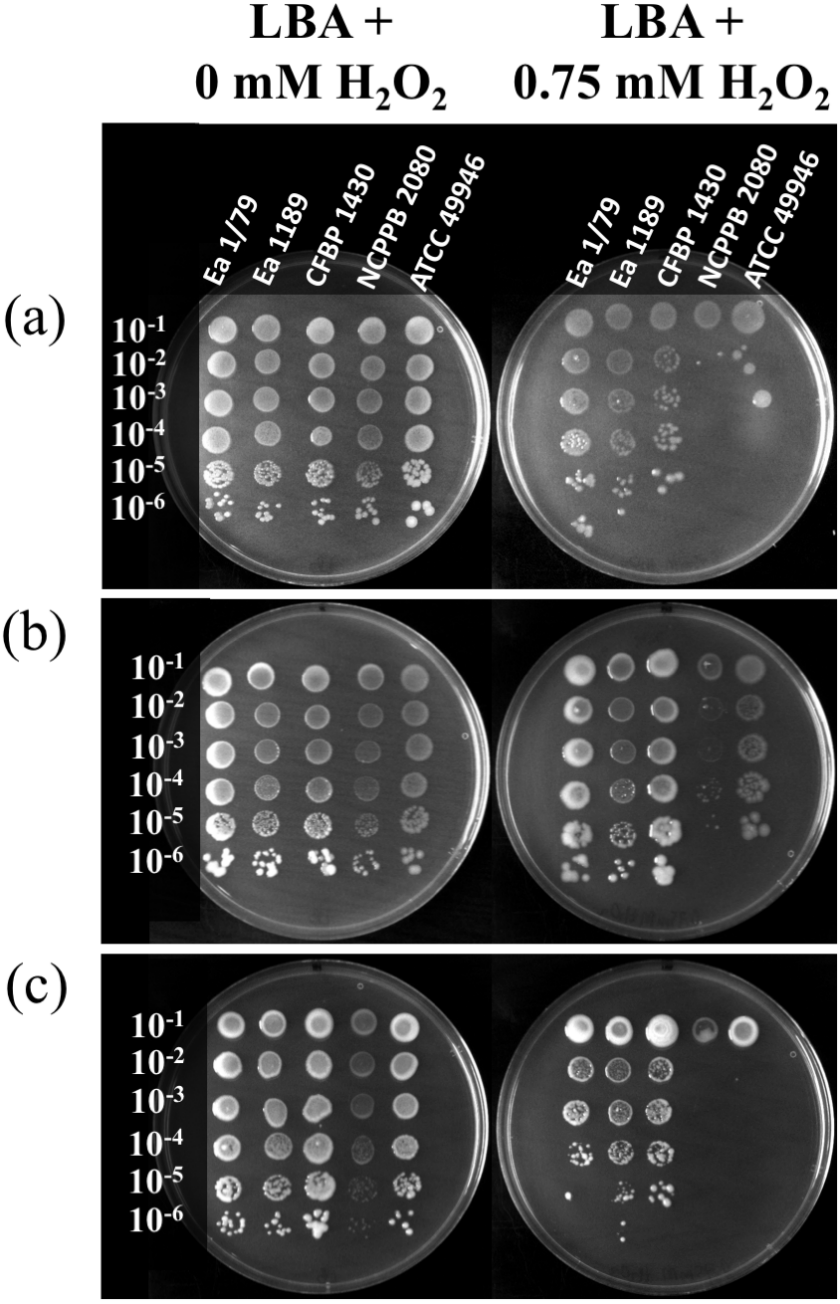
Quantification of the oxidative stress resistance of *E. amylovora* at 28 °C (A), 14 °C (B) and 4 °C (C) by an agar dilution assay using LBA plates containing 0.75 mM H_2_O_2_. Plates inoculated with serial tenfold dilutions of *E. amylovora* overnight cultures were sealed, incubated at each temperature and photographed at 6, 18 and 41 dpi, respectively. Oxidative stress resistance was greater at 14 °C than at 28 °C or 4 °C. These pictures are representative of an experiment repeated twice with similar results.

Apart from producing EPSs and forming biofilms, the *E. amylovora* invasion of host tissues depends on other virulence/pathogenicity factors (Piqué et al., 2015) such as a type three secretion system (TTSS) and TTSS-related effector proteins, which are used to kill host plant cells by means of a strategy that distinguishes *E. amylovora* from other phytopathogens (Venisse, Gullner & Brisset, 2001; Venisse et al., 2003). While many plant pathogens avoid or reduce host responses via deployment of the TTSS (Grant et al., 2006), *E. amylovora* uses the same mechanism to provoke an oxidative burst, which is used to kill plant cells (Venisse, Gullner & Brisset, 2001; Venisse et al., 2003). Accordingly, antioxidant mechanisms are required to face the reactive oxygen species (ROS) released by host cells. In fact, catalases have been reported as virulence factors in *E. amylovora* (Santander, Figàs-Segura & Biosca, 2017). Our results indicate that *E. amylovora* resistance to oxidative stress is also temperature dependent, with resistance to H_2_O_2_ being enhanced at 14 °C (Fig. 7B), with respect to 28 °C (Fig. 7A) and 4 °C (Fig. 7C). Interestingly, the production of EPSs was also more efficiently induced at 14 °C than at 28 °C (Figs. 5A–5F). Although, the role of these macromolecules as protecting agents against H_2_O_2_ has been discussed (Venisse et al., 2003), they are induced by oxidative stress (Santander, Figàs-Segura & Biosca, 2017), suggesting their possible contribution to the protection against other ROS, or toxins secreted by plant cells, which indirectly might cause oxidative stress.

It is remarkable that, despite the clear enhancement of H_2_O_2_ sensitivity at 4 °C and 28°C (Figs. 7A–7C), all the *E. amylovora* strains were able to cause regular fire blight symptoms in immature fruits (Figs. 1A–1G), with the strains which were more sensitive to oxidative stress (NCPPB 2080 and 49946) (Figs. 7A–7C) being similarly or even more virulent than some of the strains showing better resistance to H_2_O_2_. A strain-dependent production of factors reducing the effects of ROS on bacterial cells, such as the lipopolysaccharide (Berry et al., 2009) or cold shock proteins (Burbank & Stenger, 2016), probably also contributes to the phenotypes observed. Siderophore secretion has also been related to oxidative stress protection in *E. amylovora* (Dellagi et al., 1998; Dellagi et al., 1999; Venisse et al., 2003). However, although an enhanced siderophore production was observed at 28 °C, the higher resistance to H_2_O_2_ occurred at 14 °C, suggesting that other factors are involved in this stress resistance under the assayed conditions.

### Starvation at 14 °C favors the persistence in a culturable state, while the VBNC response is enhanced at 28 °C and, to a lesser extent, at 4 °C

**Figure 8:**
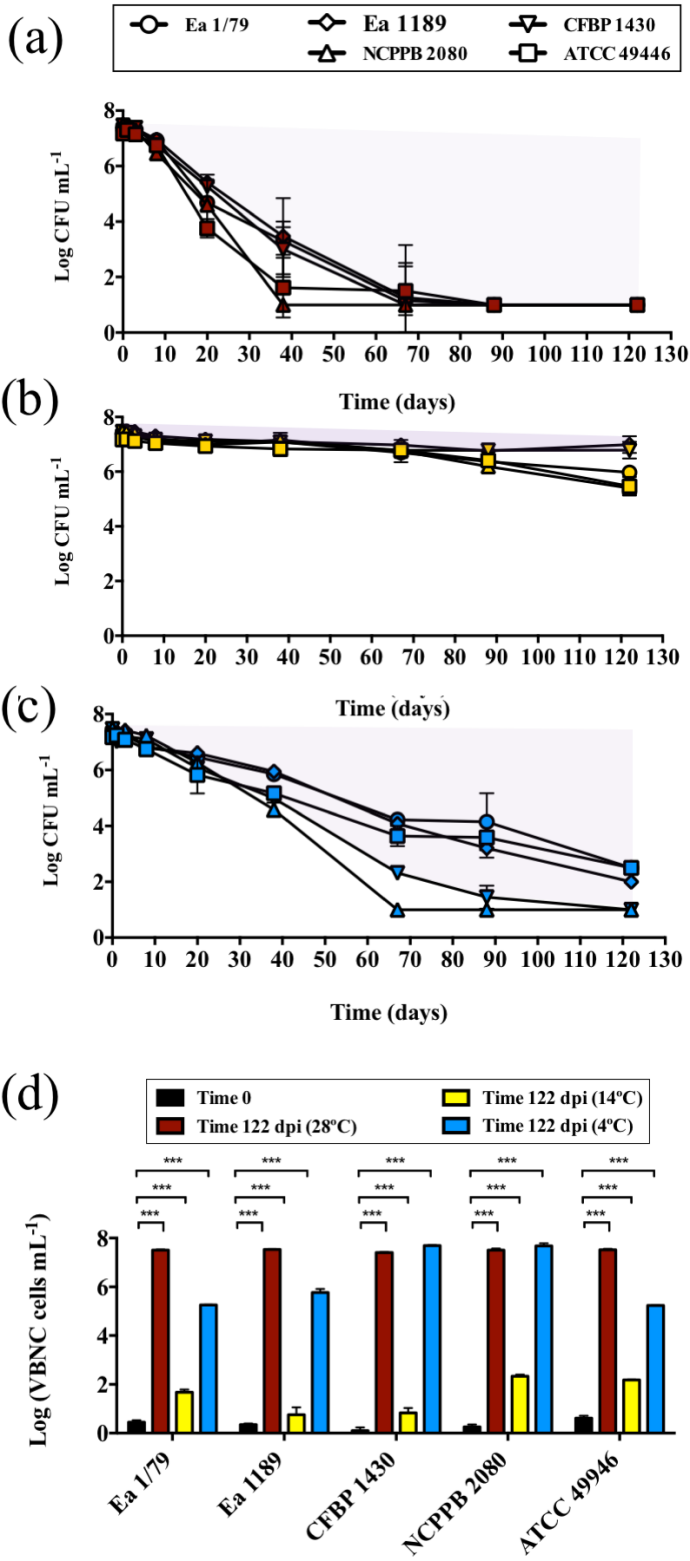
Analysis of *E. amylovora* culturable and VBNC cell populations during a long-term starvation period (122 days) at 28 °C (A), 14 °C (B) and 4 °C (C). Culturable cell counts are represented with symbols (A–C). Purple areas indicating VBNC cell subpopulations are drawn (A–C). Given the similar trends in the VBNC induction of all the assayed *E. amylovora* strains, only results corresponding to strain CFBP 1430 are shown. VBNC cell populations at times 0 and 122 dpi are summarized in (D). Vertical lines indicate the SD, and asterisks denote statistically significant differences between temperatures (***, *p* < 0.001). These data are representative of an assay performed with three microcosms per strain and temperature, inoculated in independent experiments.

As part of its life cycle, *E. amylovora* has to deal with periods of starvation, either within host cankers, as an epiphyte, inside vectors, or when spread by rainwater, etc. (Thomson, 2000; Santander, Oliver & Biosca, 2014). In a previous work we showed the temperature-dependent nature of *E. amylovora* responses to nutrient limitation (Santander, Oliver & Biosca, 2014). However, the effect of temperatures below 18 °C on starvation responses had not yet been assessed. Results in this work demonstrated the modulation of *E. amylovora* long-term starvation responses by temperature (Figs. 8A–8D). A progressive drop of culturability over time was observed at 28 °C (Fig. 8A) and, to a lesser extent, at 4 °C (Fig. 8C). This phenotype, however, was not observed at 14 °C, a temperature at which *E. amylovora* cells developed a characteristic starvation-survival response, with culturability values being similar to those at initial time, with only a slight reduction of culturability at final time (Fig. 8B).

Flow cytometry analyses showed a temperature-dependent loss of viability (*p* < 0.05) between initial and final (122 days) times, which was greater at 28 °C, followed by 14 °C, but nonsignificant at 4 °C (Fig. S2). Nevertheless, compared to culturable cell counts, this decrease of viability was very reduced, indicating that the drop in culturability observed at 28 °C and 4 °C actually corresponded to a progressive entry into the VBNC state, with the number of VBNC cells at the end of the experiment at 14 °C being reduced compared to that observed at 28 °C or 4 °C (Fig. 8D). The flow cytometric analysis of *E. amylovora* total cell populations also revealed a certain cell lysis at final time, which was greater at 28 °C, followed by 14 °C and 4°C (*p* < 0.01) (Fig. S2).

As demonstrated in previous works, *E. amylovora* cells starved for long periods remain fully virulent while culturable (Santander et al., 2012; Santander, Oliver & Biosca, 2014). Starved cells entering the VBNC state, however, lose their ability to cause disease in immature fruits, although virulence can be recovered by passage through a susceptible host (Santander et al., 2012; Ordax et al., 2015). Interestingly, the loss of culturability characteristic of the VBNC state has been linked to a reduced resistance to oxidative stress (Oliver, 2010), and *E. amylovora* mutants defective in the sigma factor RpoS, which regulates a variety of antioxidant enzymes, or mutants lacking catalase activity, show an enhanced starvation-induced VBNC response (Santander et al., 2014; Santander, Figàs-Segura & Biosca, 2017), which agrees with results obtained in this work.

Temperature dependent alternating periods of VBNC and starvation-survival responses are characteristic stages of the life cycle of many non-obligated pathogens, and determine the seasonal incidence of the disease they cause (Armada et al., 2003; Caruso et al., 2005; Smith & Oliver, 2006; Vattakaven et al., 2006; Ayrapetyan et al., 2015). The knowledge of the roles that these survival responses play in the *E. amylovora* life cycle, or how environmental temperatures affect such responses may, indeed, allow for an improvement in control and/or prevention measures against fire blight.

### Starvation induces morphological changes in a temperature-dependent manner

Similar to other bacteria that change their shape during their life cycle (Morita, 1997; Álvarez, López & Biosca, 2008; Chen et al., 2009; Oliver, 2010; Yang, Blair & Salama, 2016), *E. amylovora* responds to starvation with a reduction in cell size (dwarfing) and the acquisition of rounded shapes, probably via the degradation of endogenous material, cell walls and envelopes through the formation of surface vesicles (Santander, Oliver & Biosca, 2014). In many pathogens, morphological changes occurring under starvation conditions are dependent on temperature (Chen et al., 2009; Yang, Blair & Salama, 2016), however, the influence of this environmental factor on *E. amylovora* starved cells had not yet been explored.

**Figure 9:**
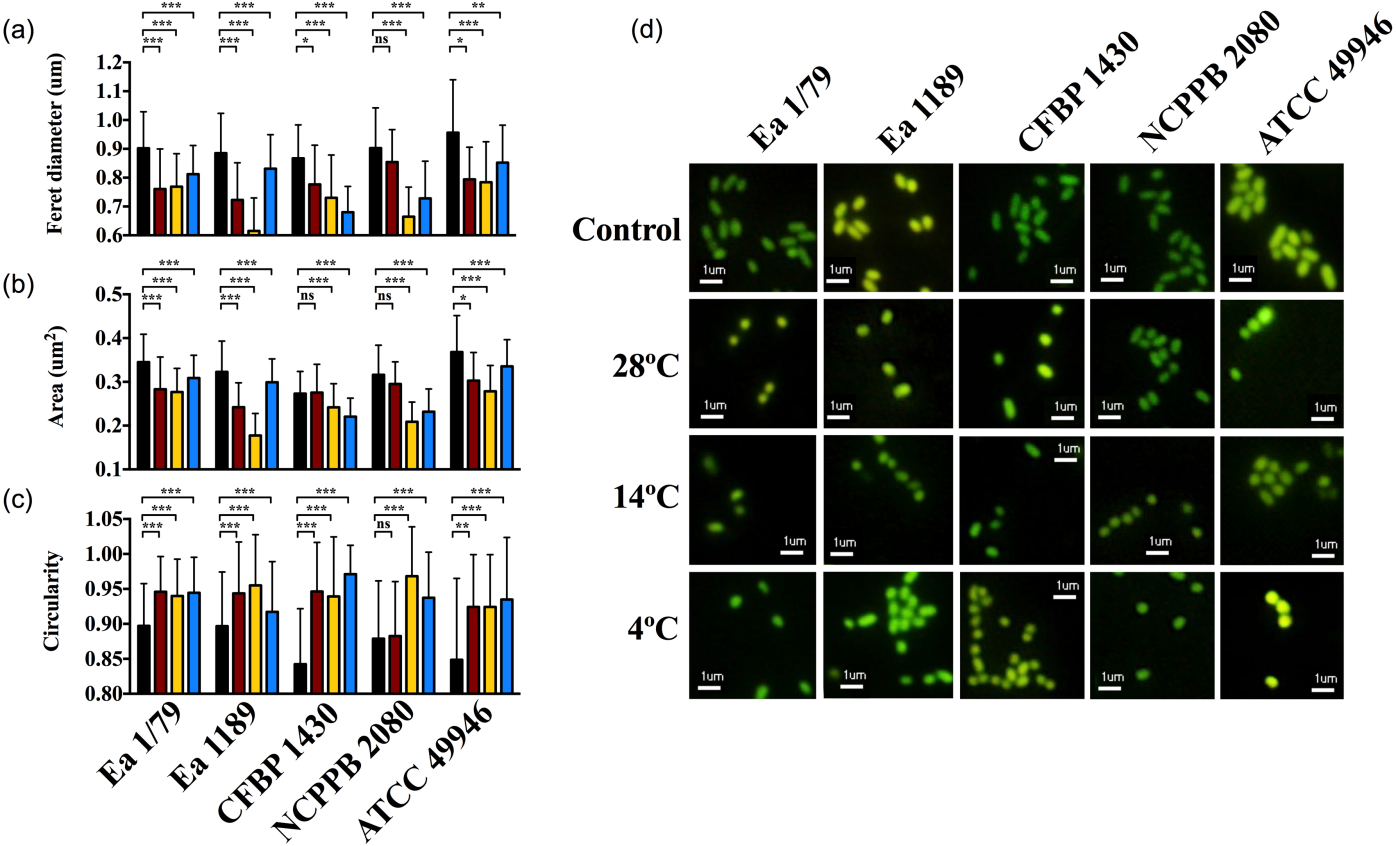
*E. amylovora* morphological changes induced by starvation at different temperatures. Variations in cell length (Feret diameter) (A), area (B) and circularity (C) (average values of more than 100 measurements per microcosm) in cells at time 0 (black columns) with respect to cells starved for 122 days at 28 °C (red columns), 14 °C (yellow columns) and 4 °C (blue columns). Representative pictures of the previous data (D). Asterisks above horizontal lines denote statistically significant differences between two conditions (*, *p* < 0.05; **, *p* < 0.01; ***, *p* < 0.001; ns, non-significant differences). These data are representative of one of three assays (microcosms) per strain and temperature, inoculated in independent experiments and which showed similar results.

At time 0, *E. amylovora* cells showed a typical rod shape (Figs. 9A–9D). However, in general, prolonged exposure to starvation provoked the acquisition of rounded shapes and dwarfing (Figs. 9A–9D). Except for strain NCPPB 2080, the acquisition of rounded shapes in response to oligotrophy was observed at all the temperatures assayed (*p* < 0.05). However, a strong effect of incubation temperature on the cell size of long-term starved *E. amylovora* cells was observed (*p* < 0.001). Most strains experienced enhanced dwarfing at 14 °C, while the effect of the other temperatures on size reduction varied depending on the assayed strain (Figs. 9A–9D).

The reduction of bacterial cell size and the acquisition of coccoid shapes have been related to the optimization of energetic resources in starved cells, but also as a strategy to avoid predation by protists (Byrd, 2000; Nyström, 2004). Moreover, for some pathogens, morphological transitions occur in parallel to the colonization of different host tissues or cell types, transmission between hosts and/or shifts to environmental reservoirs (Yang, Blair & Salama, 2016). *E. amylovora* cells during infections are rod-shaped, similar to what occurs during growth in liquid medium. Our results indicate that prolonged starvation conditions such as in reservoirs outside the host, or also within the host in cankers, would induce size reduction and the adoption of circular shapes, with this response being favored at temperatures enhancing the persistence of the pathogen in a culturable and pathogenic state, such as 14 °C and also 20 °C (Santander, Oliver & Biosca, 2014).

Taken together the results in this work and a previous one (Santander, Oliver & Biosca, 2014), it can be deducted that temperatures ranging from 14 °C to 20 °C would favor the persistence of *E. amylovora* in the environment in a culturable and pathogenic state, reducing cell size and adopting coccoid shape in response to environmental starvation. Moreover, temperate temperatures would coincide with those ensuring a greater abundance of pollinating insects (or other biotic vectors), and contributing to the epiphytic growth on blossoms, motility, chemotactic responses, and fire blight symptom development when inside host tissues (Raymundo & Ries, 1980; Raymundo & Ries, 1981; Thomson, 2000; Pusey & Curry, 2004; Van der Zwet, Orolaza-Halbrendt & Zeller, 2012; Santander, Oliver & Biosca, 2014). However, lower and higher temperatures in other periods of the year would induce the development of the VBNC response (e.g., inside host cankers) in parallel to the loss of pathogenicity in immature fruits (Santander et al., 2012), but with an enhanced environmental stress resistance (Oliver, 2010; Nowakowska & Oliver, 2013). Nevertheless, such VBNC cells can recover their pathogenicity under appropriate conditions (e.g., by passage through pear plantlets) (Santander et al., 2012; Ordax et al., 2015).

Overall, this study provides, for the first time, new data on *E. amylovora* psychrotrophic adaptations, evidencing its pathogenic potential, production of different virulence factors, and survival at a wide range of environmental temperatures (4 °C–28 °C) shedding light on scarcely known aspects of the biology of this pathogen. This knowledge provides new and valuable information for the improvement of an integrated management of fire blight disease.

##  Supplemental Information

10.7717/peerj.3931/supp-1 Figure S1 Virulence assays in immature pears (cv. Devoe) with the *E. amylovora* strain CFBP 1430 at 28 °C (A), 14 °C (B) and 4 °C (C) Graphs show the percentage of symptomatic fruits over time. Representative pictures of fruits showing fire blight symptoms at the end of the experimental period (28 °C, 10 dpi; 14 °C, 15 dpi; 4 °C, 51 dpi) are represented besides the corresponding graph. Vertical lines indicate the SD.

10.7717/peerj.3931/supp-2 Figure S2 Flow cytometry analysis of total and viable cell counts in *E. amylovora* starved cells at times 0 and 122 dpi Strains Ea 1/79 (A), Ea 1189 (B), CFBP 1430 (C), NCPPB 2080 (D) and ATCC 49946. Vertical lanes indicate the SD. An ANOVA analysis of data revealed an effect of temperature on viability (*p* < 0.05) and total cell integrity (total cell counts) (*p* < 0.01).

10.7717/peerj.3931/supp-3 Data S1 Raw data used in Fig. 1

10.7717/peerj.3931/supp-4 Data S2 Raw data used in Fig. 2

10.7717/peerj.3931/supp-5 Data S3 Raw data used in Fig. 3

10.7717/peerj.3931/supp-6 Data S4 Raw data used in Fig. 4

10.7717/peerj.3931/supp-7 Data S5 Raw data used in Fig. 5

10.7717/peerj.3931/supp-8 Data S6 Raw data used in Fig. 6

10.7717/peerj.3931/supp-9 Data S7 Raw data used in Fig. 8

10.7717/peerj.3931/supp-10 Data S8 Raw data used in Fig. 9

## Additional Information and Declarations

### Data Availability

The following information was supplied regarding data availability: The raw data from representative experiments used to create the figures in the paper have been provided as Supplemental Files.
