# Supplementary figures and images for: Erwinia amylovora psychrotrophic adaptations: evidence of pathogenic potential and survival at temperate and low environmental temperatures
Source: PeerJ. 2017 Oct 26;5:e3931. doi: 10.7717/peerj.3931 (PMC5660878; doi:10.7717/peerj.3931)

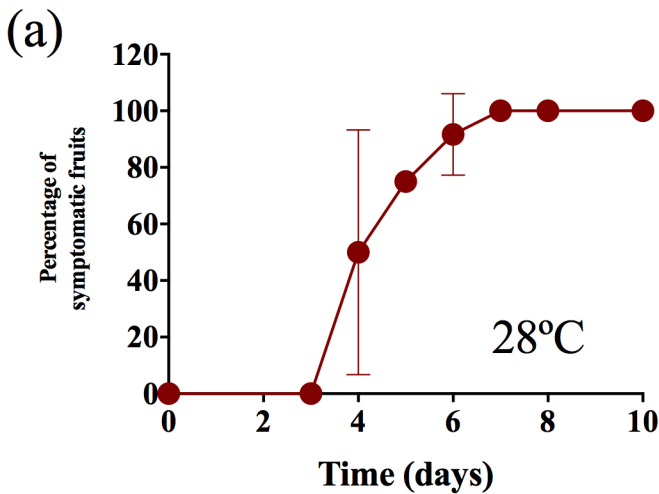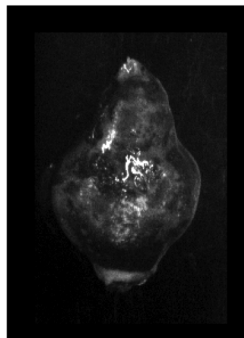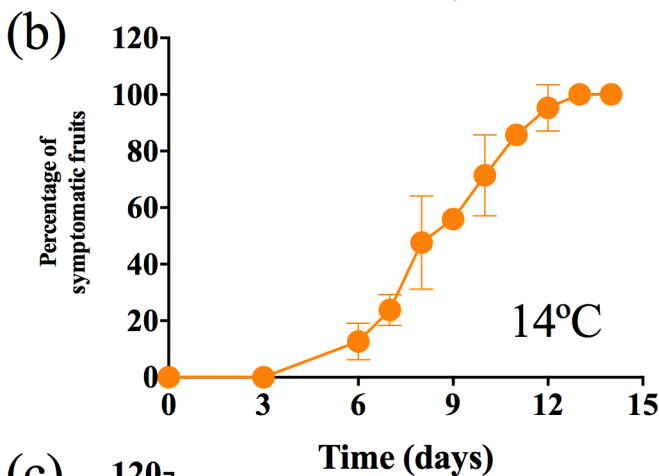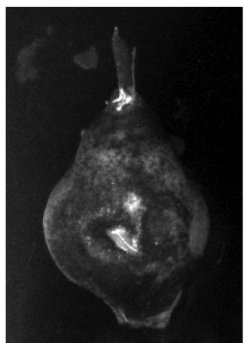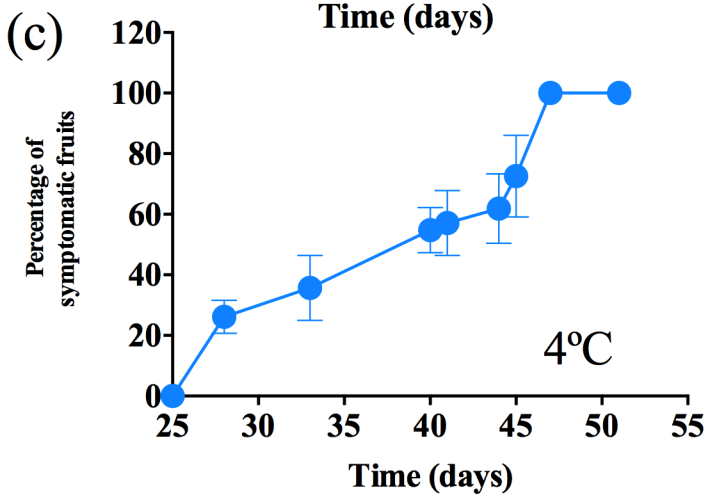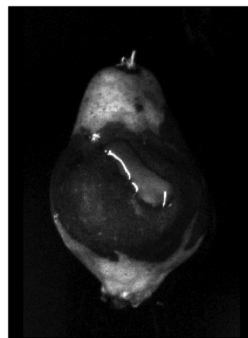

Supplement: Figure S1 — Graphs show the percentage of symptomatic fruits over time. Representative pictures of fruits showing fire blight symptoms at the end of the experimental period (28 °C, 10 dpi; 14 °C, 15 dpi; 4 °C, 51 dpi) are represented besides the corresponding graph. Vertical lines indicate the SD. [file peerj-05-3931-s001.pdf]

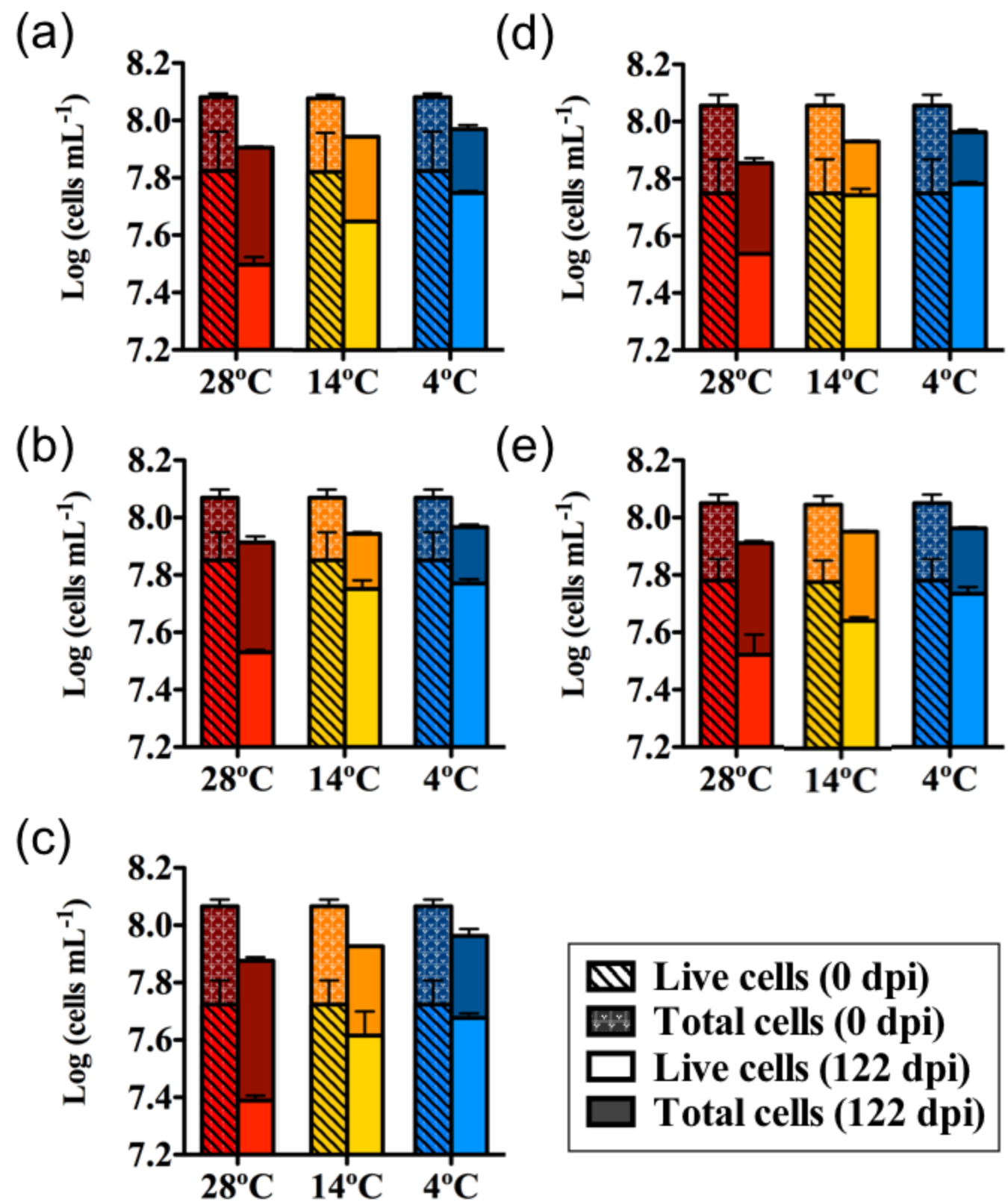

Supplement: Figure S2 — Strains Ea 1/79 (A), Ea 1189 (B), CFBP 1430 (C), NCPPB 2080 (D) and ATCC 49946. Vertical lanes indicate the SD. An ANOVA analysis of data revealed an effect of temperature on viability (p < 0.05) and total cell integrity (total cell counts) (p < 0.01). [file peerj-05-3931-s002.pdf]
